# Development of a sodium alginate-silver/zinc oxide – cinnamaldehyde nanocomposite film for coating chicken meat and evaluation of meat safety

**DOI:** 10.1039/d6ra01488g

**Published:** 2026-03-30

**Authors:** K. A. Deepika Roy, Shivaprasad Shivappa Desai, Padikkamannil Abishad, Rahul Krishnan, Valil Kunjukunju Vinod, J. J. Sruthimol, Anjineyulu Kothakotta, Sukhadeo Baliram Barbuddhe, Deepak Bhiwa Rawool, Jess Vergis

**Affiliations:** a College of Veterinary and Animal Sciences, Kerala Veterinary and Animal Sciences University (KVASU) Pookode 673 576 Wayanad India itzjessvergis@gmail.com +91-9446355683; b Department of Aquatic Animal Health Management, Kerala University of Fisheries and Ocean Studies Panangad-682 506 Kochi India; c CSIR – National Institute for Interdisciplinary Science and Technology Industrial Estate P.O.-695 019 Thiruvananthapuram India; d ICAR-National Meat Research Institute Chengicherla, Boduppal Post-500 092 Hyderabad India

## Abstract

Developing sustainable and edible active packaging materials is critical to mitigate microbial contamination and enhance food safety. This study developed a biodegradable and edible active film incorporating green-synthesized silver-zinc oxide nanocomposites (Ag/ZnO NCs) entrapping cinnamaldehyde (Ag/ZnO–N) in a food-grade alginate matrix for preservation of chilled chicken meat. The Ag/ZnO–N exhibited antibacterial activity against multi-drug-resistant (MDR) enteroaggregative *Escherichia coli*, *Salmonella* spp., and methicillin-resistant *Staphylococcus aureus*, with a minimum inhibitory concentration and minimum bactericidal concentration (MBC) of 7.80 and 62.50 µg mL^−1^, respectively. UV-vis and Fourier-transform infra-red spectroscopic analyses confirmed nanoparticle formation and cinnamaldehyde entrapment, while X-ray diffraction and scanning electron microscopy revealed polycrystalline morphology with reduced lattice crystallinity. The NCs exhibited minimal cytotoxicity to Vero cells (83.35% viability at 10^−5^ mg mL^−1^). Alginate (5%) films incorporating Ag/ZnO–N at MBC levels demonstrated enhanced surface roughness by atomic force microscopy, functional group integration, and potent antioxidant capacity (74.50 ± 0.14% ABTS^˙+^ and 8.38 ± 1.18% DPPH radical scavenging). The films were non-inhibitory to commensal microflora and exhibited significant antibacterial efficacy against MDR pathogens. In an *ex vivo* study on vacuum-packed chicken meat stored for 15 days under chilling conditions, the film significantly (*P* < 0.05) reduced aerobic plate, psychrotrophic, *E. coli*, and *S. aureus* counts, while *Salmonella* spp. were undetected. Lipid oxidation remained negligible and inductively coupled plasma mass spectrometry confirmed the absence of Ag^+^ and Zn^2+^ migration. These findings demonstrate that alginate-Ag/ZnO–N film offers a safe, edible, functional, and environmentally sustainable biomaterial platform for meat preservation, supporting circular bioeconomy-driven food systems.

## Abstract 2

Developing sustainable and edible active packaging materials is critical to mitigate microbial contamination and enhance food safety.

## Graphical abstract

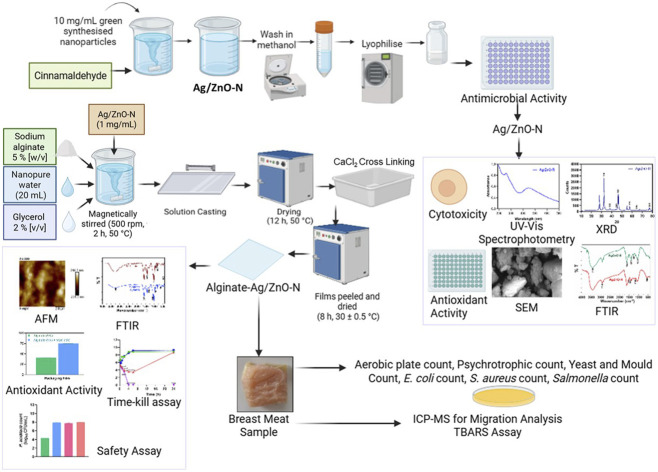

## 1. Introduction

Food safety remains a critical global challenge, with unsafe food responsible for illness in one out of every ten individuals and contributing to an estimated 33 million lost years of healthy living annually, according to the World Health Organization.^1^ Bacterial pathogens such as *Escherichia coli*, *Salmonella* spp., and *Staphylococcus aureus* account for nearly two-thirds of global food-borne infections, posing major obstacles to achieving food safety, particularly in developing countries.^2,3^ The increasing prevalence of drug resistance among these pathogens further complicates treatment and amplifies risks to food safety.^4^

Antimicrobial resistance (AMR) is recognized as one of the foremost public health threats of the 21st century, causing an estimated 1.27 million deaths annually and undermining progress toward multiple United Nations Sustainable Development Goals.^4,5^ With ongoing economic growth, changing consumption patterns, and population expansion, global food production is expected to increase by 50–70% between 2010 and 2030. Correspondingly, antimicrobial use in food production sectors such as meat, milk, and eggs is predicted to rise at a comparable rate.^4^ In the context of a declining antibiotic discovery pipeline, strategies such as drug repurposing and the development of innovative drug delivery systems are urgently needed to enhance antimicrobial efficacy and mitigate the AMR crisis.^5,6^

Food packaging plays a pivotal role in ensuring food safety and quality by protecting food products from environmental factors, microbial contamination, and spoilage during post-harvest handling, storage, distribution, and transportation.^7^ However, the extensive use and poor disposal of single-use plastic packaging materials have led to significant environmental concerns due to their non-biodegradable nature. The growing detection of microplastics and nano-plastics in food products has further intensified public health and ecological concerns, emphasizing the need for sustainable, circular economy-based packaging solutions.^8^ Among natural antimicrobial agents, essential oils (EOs) are a promising class of plant-derived bio-actives with wide-ranging antimicrobial and functional properties suitable for food applications.^9^ Cinnamaldehyde, a potent constituent of cinnamon (*Cinnamomum* spp.) EO, exhibits antibacterial, antifungal, insecticidal, anti-cancer, and anti-hyperglycemic properties, making it an attractive natural preservative for food products.^10,11^ However, its high volatility, hydrophobicity, and tendency to interact adversely with food matrices can lead to reduced stability and undesirable organoleptic effects, limiting its direct application in foods. Nanoencapsulation of cinnamaldehyde can overcome these drawbacks by enhancing its stability, controlling its release, and protecting it from environmental degradation, thereby improving its antimicrobial efficacy in food systems.^12^

In recent years, nanotechnology has gained prominence in food preservation and safety due to its ability to enhance antimicrobial delivery, extend shelf life, and prevent quality deterioration during storage.^9,13^ Green synthesis methods for nanoparticle (NP) production have been particularly emphasized as sustainable alternatives to conventional chemical synthesis, owing to their lower energy requirements, environmental safety, and reduced toxicity.^14^ Facile green synthesis of Ag/ZnO nanocomposites (NCs) exhibiting potent antibacterial activity against multi-drug-resistant (MDR) strains of *E. coli*, *Salmonella* Enteritidis, *S*. Typhimurium, and methicillin-resistant *S. aureus* (MRSA) was reported earlier.^15,16^ In this context, the incorporation of EO constituents such as cinnamaldehyde into green-synthesized NPs offers a sustainable approach for developing biodegradable antimicrobial food packaging materials.^17,18^ Active packaging systems are increasingly explored for their potential to inhibit microbial growth and extend the shelf life of perishable food products.^7,19^ However, the integration of fully eco-friendly components-particularly green-synthesized Ag/ZnO NCs entrapped with volatile plant phytochemicals-remains limited in the literature. Therefore, this study aimed to develop an active, biodegradable antimicrobial food packaging material by incorporating green-synthesized cinnamaldehyde-entrapped Ag/ZnO NCs into an alginate polymer matrix, and to evaluate its efficacy in maintaining the microbial quality of chilled chicken breast meat.

## 2. Experimental

### 2.1. Bacterial strains and phytochemicals

Previously characterized multidrug-resistant (MDR) pathogenic strains, including MRSA (*n* = 3; Sa1, Sa2, Sa3), *S. enterica* serovar typhimurium (*n* = 3; ST1, ST2, ST3), *S. enterica* serovar enteritidis (*n* = 3; S1, S2, S3), and enteroaggregative *E. coli* (EAEC; *n* = 3; E1, E2, E3), were employed in this study. In addition, standard strains of commensal gut bacteria- *Pediococcus acidilactici* ATCC 8042, *Lactobacillus acidophilus* MTCC 10307, and *L. plantarum* MTCC 5690- were included for comparative evaluation. Cinnamaldehyde (≥95% purity; Sigma-Aldrich, St. Louis, MO, USA), previously identified and characterized, was utilized for nanoparticle entrapment.^20,21^

### 2.2. Green synthesis of Ag/ZnO NCs

The green synthesis of Ag/ZnO NCs was performed,^14^ employing the methanolic extract of *Curcuma longa* as a biogenic reducing and stabilizing agent. Briefly, 20 mL of *C. longa* methanolic extract was gently mixed with 48 mL of 0.10 M zinc nitrate dihydrate and 32 mL of 0.10 M silver nitrate aqueous solutions. The mixture was subjected to magnetic stirring at 300 rpm and maintained at 60 °C for 24 h. The gradual change in the solution colour from light yellow to dark brown indicated the successful formation of Ag/ZnO NCs.^16^ The formation of the nanomaterial was subsequently confirmed using UV-vis spectroscopy.

### 2.3. Entrapment of Ag/ZnO NCs with cinnamaldehyde (Ag/ZnO–N)

**Fig. 1:**
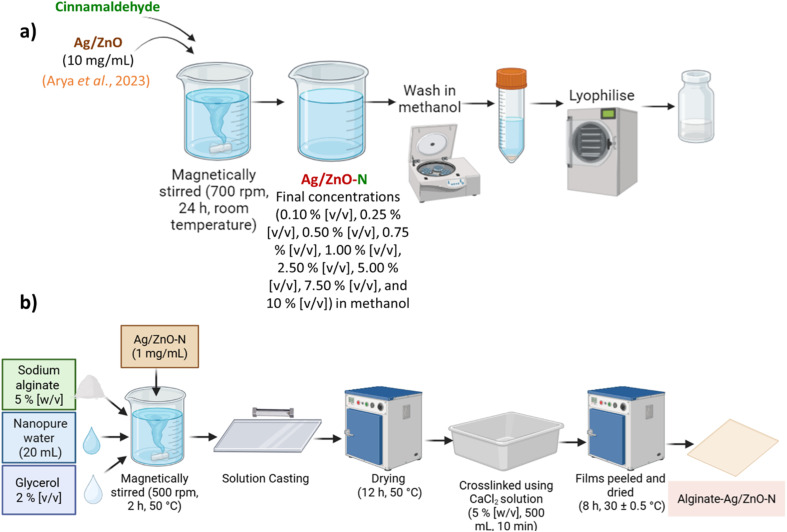
Schematic representation of (a) Ag/ZnO–N synthesis and (b) alginate-Ag/ZnO–N film preparation.

A methanolic suspension of Ag/ZnO NCs (10 mg mL^−1^) was continuously agitated with cinnamaldehyde at room temperature for 24 h at 700 rpm using a magnetic stirrer (Dan Logitech Instruments Pvt. Ltd, India). The mixture was prepared to achieve final cinnamaldehyde concentrations of 0.10, 0.25, 0.50, 0.75, 1.00, 2.50, 5.00, 7.50, and 10.00% (v/v) in methanol (Fig. 1a).

### 2.4. Characterisation of Ag/ZnO–N

The physicochemical characterization of Ag/ZnO–N was performed using UV-vis spectrophotometry, Fourier Transform Infrared Spectroscopy (FTIR), X-ray Diffraction (XRD), and Scanning Electron Microscopy (SEM). UV-vis spectral analysis of Ag/ZnO–N was carried out in the wavelength range of 200–800 nm using a UV-vis spectrophotometer (Thermo Fisher Scientific, USA), with plain Ag/ZnO NCs and cinnamaldehyde serving as controls. The surface functional groups associated with Ag/ZnO–N were identified using FTIR spectroscopy (PerkinElmer C94012, USA) within the scanning range of 400–4000 cm^−1^ at a spectral resolution of 4 cm^−1^. The crystalline structure of the NCs was analyzed by powder XRD using a Bruker D8 Advance diffractometer (USA) equipped with a Cu Kα radiation source (*λ* = 1.54060 Å), operated at 40 keV and 40 mA, with a step size of 0.02°. Surface morphology was examined using scanning electron microscopy (SEM; JEOL JSM-6390LV, Japan). The elemental composition of Ag/ZnO–N was further determined by energy-dispersive X-ray (EDX) analysis coupled with the SEM.

### 2.5. *In vitro* antimicrobial activity of Ag/ZnO–N against MDR test pathogens

The antimicrobial activity of Ag/ZnO–N was evaluated by determining the *in vitro* minimum inhibitory concentration (MIC) and minimum bactericidal concentration (MBC) against MDR isolates of EAEC, non-typhoidal *Salmonella* (NTS), and MRSA, following the guidelines of the Clinical and Laboratory Standards Institute.^22^ The MIC was defined as the lowest concentration of Ag/ZnO–N that inhibited bacterial growth, as indicated by the absence of resazurin dye reduction.

For MBC determination, 10 µL aliquots from wells exhibiting no visible colour change were inoculated onto respective selective agar plates and incubated under appropriate conditions. The MBC was recorded as the lowest concentration of Ag/ZnO–N that resulted in ≥99.9% bacterial kill on selective media.

Among the various concentrations of Ag/ZnO–N, the formulation exhibiting optimal antibacterial activity with the minimal incorporation of cinnamaldehyde was selected for further experimentation. The Ag/ZnO NCs containing 0.10% cinnamaldehyde demonstrated the lowest MIC values and were therefore used in subsequent analyses.

### 2.6. *In vitro* antioxidant activity of Ag/ZnO–N

The *in vitro* antioxidant activity of Ag/ZnO–N was evaluated using the 2,2′-azinobis (3-ethylbenzothiazoline-6-sulfonic acid) (ABTS^˙+^)-based radical scavenging assay.^23,24^ Briefly, a stock solution of ABTS (7 mM; Sisco Research Laboratories Pvt. Ltd, India) and potassium persulfate (140 mM; SRL) was incubated at room temperature in the dark for 15 h to generate the ABTS^˙+^ radical. The resultant ABTS^˙+^ solution was then diluted with phosphate buffer (10 mM, pH 7.4; HiMedia Laboratories Pvt. Ltd, India) to obtain an absorbance of 0.70 at 734 nm. Gradually increasing concentrations of Ag/ZnO–N (0, 20, 40, 60, 80, and 100 µg mL^−1^) were prepared, with equivalent concentrations of ascorbic acid serving as the standard antioxidant. The mixtures were incubated aerobically in the dark at 37 °C for 20 min, and the absorbance was measured at 734 nm. The percentage radical scavenging activity was calculated using the formula (*A*_Control_ − *A*_Test_)/(*A*_Control_) × 100, wherein *A*_Control_ represents the absorbance of the control, whereas *A*_Test_ denotes the absorbance of test samples.^25^

### 2.7. *In vitro* cell viability assay

The *in vitro* safety of Ag/ZnO–N on eukaryotic cells was evaluated using a Vero cell line-based cytotoxicity assay,^26^ following the protocol of the MTT cell proliferation assay kit (Sigma-Aldrich, USA). Cell monolayers were obtained by pre-culturing Vero cells in Dulbecco's Modified Eagle Medium (DMEM; pH 7.2) supplemented with 10% foetal bovine serum (FBS; Gibco, USA). The cells were seeded into 96-well microtitre plates at a density of 1 × 10^4^ cells per well and incubated overnight at 37 ± 0.5 °C. Thereafter, the cells were exposed to serially decreasing concentrations of Ag/ZnO–N (10^−1^ to 10^−5^ mg mL^−1^) dispersed in DMEM, while cells maintained in fresh DMEM served as the negative control. Following 24 h incubation at 37 °C in a 5% CO_2_ atmosphere, the supernatant was removed and 50 µL of MTT reagent along with culture medium was added to each well and incubated for 3 h at 37 °C. Subsequently, 100 µL of MTT solvent was added to dissolve the formazan crystals, and absorbance was recorded at 550 nm using a microplate reader. The cytotoxicity (%) was calculated using the formula (*A*_Control_ − *A*_Test_)/(*A*_Control_) × 100, where *A*_Control_ represents the absorbance of untreated cells and *A*_Test_ corresponds to the absorbance of treated cells.^27^

### 2.8. Preparation of active antimicrobial packaging film

Antimicrobial packaging films were prepared using sodium alginate (SA; Loba Chemie, India) following the method described earlier,^28^ with certain modifications. Briefly, a 5% (w/v) SA solution was prepared in nanopure water and plasticized with 2% (v/v) glycerol. Desired volumes of Ag/ZnO–N suspension (1 mg mL^−1^ in nanopure water) were incorporated into 10 mL of the alginate solution to achieve final concentrations corresponding to the MIC and MBC levels of Ag/ZnO–N. The mixture was stirred continuously at 50 °C for 2 h using a magnetic stirrer (500 rpm) to obtain a homogeneous film-forming solution. The solution was then cast on a sterile glass plate (40 × 18 cm) using the 400 µm edge of a four-sided film applicator (coating width: 160 mm; Raj Scientific Enterprises Pvt. Ltd, India) and dried at 50 °C for 12 h. The resulting SA films were immersed in 5% (w/v) calcium chloride solution (500 mL) for 10 min to induce cross-linking, after which they were peeled off and air-dried at room temperature for 8 h (Fig. 1b). The dried alginate-Ag/ZnO–N films were stored in sterile sample bags (6″ × 13″; Tarsons Pvt. Ltd, India) at ambient temperature until further analysis. A plain alginate film without Ag/ZnO–N served as the control.

### 2.9. Assessment of the effect of alginate-Ag/ZnO–N films on commensal gut microflora

The potential impact of the alginate-Ag/ZnO–N films on commensal gut microflora was evaluated to ensure their biosafety, as described.^25^ Briefly, 100 mg of alginate-Ag/ZnO–N films containing Ag/ZnO–N at MIC and MBC concentrations were co-incubated separately with cultures of *Lactobacillus acidophilus* MTCC 10307, *L. plantarum* MTCC 5690, and *Pediococcus acidilactici* ATCC 8042 (1 × 10^7^ CFU mL^−1^) in 5 mL of de Man–Rogosa–Sharpe (MRS) broth. Growth controls (untreated bacterial cultures) and negative controls (sterile MRS broth) were maintained in parallel. Following incubation at 37 °C for 48 h, 10 µL aliquots were drawn from each culture and plated onto MRS agar.^29^ After incubation at 37 °C for 48 h, colony-forming units (CFU) were enumerated, and results were expressed as log_10_ CFU mL^−1^.^24,25^

### 2.10. *In vitro* dose- and time-dependent growth kinetics of MDR-test bacterial strains treated with alginate-Ag/ZnO–N films

The antibacterial efficacy of the alginate-Ag/ZnO–N films against MDR isolates of EAEC, *S*. Typhimurium, *S*. Enteritidis, and MRSA was evaluated through an *in vitro* dose- and time-dependent extracellular growth kinetics assay, as described.^30^

Briefly, log-phase cultures of each bacterial strain grown in nutrient broth were centrifuged at 10 000 × *g* for 10 min. The supernatant was discarded, and the bacterial pellet was resuspended in sterile phosphate-buffered saline (PBS; pH 7.4) to achieve a turbidity equivalent to the 0.50 McFarland standard (≈1.5 × 10^8^ CFU mL^−1^). The standardized inoculum was further diluted in cation-adjusted Mueller–Hinton (CA–MH) broth to obtain a final bacterial concentration of 1 × 10^7^ CFU mL^−1^. Subsequently, 100 mg of the alginate-Ag/ZnO–N film was co-incubated with 5 mL of the bacterial suspension at 37 °C for 24 h. Sterile CA–MH broth, untreated bacterial cultures, and plain alginate films co-incubated with bacterial inoculate served as experimental controls. Aliquots (10 µL) were collected at specific time intervals (0, 1, 2, 4, 6, 10, and 24 h), serially diluted in sterile PBS (90 µL), and plated (10 µL) on selective agar media- Eosin Methylene Blue (EMB) agar for MDR-EAEC, Xylose Lysine Deoxycholate (XLD) agar for MDR-*Salmonella*, and Baird–Parker (BP) agar for MDR-MRSA. Plates were incubated at 37 °C for 18–24 h, and bacterial colonies were enumerated to determine viable counts expressed as log_10_ CFU mL^−1^.^24,25,29^ The film concentration demonstrating superior antibacterial activity, corresponding to the MBC level of Ag/ZnO–N, was selected for subsequent analyses.

### 2.11. Characterization of alginate-Ag/ZnO–N film

The prepared alginate-Ag/ZnO–N films were characterized using FTIR, thermogravimetric analysis (TGA), derivative thermogravimetry (DTG), and atomic force microscopy (AFM). The FTIR analysis was conducted as described previously to identify functional groups and confirm nanoparticle incorporation within the alginate matrix. The thermal stability of the films was assessed through TGA–DTG analysis using a simultaneous thermal analyzer (SII NT TG/DTA 6200, SII Nano Technology Inc., Japan). The measurements were performed under an argon atmosphere over a temperature range of 30–700 °C at a heating rate of 10 °C min^−1^.

Surface topography and roughness were examined using AFM (Ntegra NT-MDT, UK) operated in contact mode with a scan area of 10 µm × 10 µm. Neat alginate films served as controls to evaluate the morphological and structural changes induced by nano-additive incorporation.

Barrier properties of the films were also determined. The oxygen transmission rate (OTR; cm^3^ m^−2^ d^−1^) was measured at 23 °C and 0% relative humidity using a PERME VAC-VBS Gas Permeability Tester. The water vapour transmission rate (WVTR; g m^−2^ d^−1^) was measured at 37 °C and 90 ± 2% relative humidity using a Systester WVTR C1 Water Vapour Permeability Tester.

### 2.12. *In vitro* antioxidant activity of alginate-Ag/ZnO–N film

The *in vitro* antioxidant potential of the alginate-Ag/ZnO–N films was evaluated using 2,2-diphenyl-1-picrylhydrazyl (DPPH^˙^) and 2,2′-azinobis(3-ethylbenzothiazoline-6-sulfonic acid) (ABTS^˙+^) radical scavenging assays as described,^25^ with certain modifications.

For the DPPH^˙^ assay, a methanolic DPPH solution was prepared by dissolving 4 mg of DPPH in 100 mL of methanol. Film samples (100 mg each of plain alginate and alginate-Ag/ZnO–N) were immersed in 5 mL of the DPPH solution and incubated in the dark for 6 h. The absorbance was recorded at 517 nm using a UV-vis spectrophotometer (Thermo Fisher Scientific, USA).^31^

For the ABTS^˙+^ assay, 100 mg of each film type was added to 5 mL of pre-formed ABTS^˙+^ solution (absorbance adjusted to 0.70 ± 0.02 at 734 nm) and incubated under the same conditions. The absorbance was measured at 734 nm after 6 h of incubation.^24,25^

For both assays, radical solutions without film samples served as controls. The free radical scavenging activity (%) was calculated using the formula described previously.

### 2.13. *Ex vivo* meat storage study using alginate-Ag/ZnO–N film

The *ex vivo* efficacy of the alginate-Ag/ZnO–N film in maintaining the keeping quality of chicken breast meat under chilling conditions was evaluated following the method described,^31^ with necessary modifications.

Fresh chicken breast meat was procured from a local market in Vythiri, Wayanad (11.5517° N, 76.0403° E), and stored at 4 ± 1 °C prior to experimentation. Film samples were cut into approximately 10 × 10 cm rectangular pieces. Meat samples (10 g) were aseptically wrapped with either plain alginate or alginate-Ag/ZnO–N films, while unwrapped meat served as the control. All individual samples (film-wrapped and unwrapped) were sealed in two-layer laminated pouches (polyester + low-density polyethylene, 15 × 15 cm) and vacuum-packed (Sevana Electrical Appliances, India).

Microbiological analyses were performed at three-day intervals, while migration and lipid oxidation (TBARS) assays were carried out on days 0, 7, and 14.

#### 2.13.1. Microbiological evaluation

For microbiological assays, samples were aseptically transferred to sterile stomacher bags (Tarsons Pvt. Ltd, India) containing 90 mL of 0.10% sterile peptone water (HiMedia) and homogenized for 3 min in a stomacher (BagMixer® 400, Interscience, France). The homogenates were serially diluted (10-fold, up to 10^−7^), and aliquots from appropriate dilutions were used for bacterial enumeration.

Aerobic plate counts (APCs; ISO 4833-1), psychrotrophic counts (ISO 16649-2), *Salmonella* spp. (ISO 6579), *E. coli* (ISO 6888-2) and coagulase-positive *S. aureus* (ISO 16649-2) were determined by the pour plate method, while yeast and mold counts were estimated by the spread plate method (ISO 21527).^31^

#### 2.13.2. Lipid peroxidation assay

Lipid oxidation in the meat samples was assessed by determining thiobarbituric acid reactive substances (TBARS). Meat samples (10 g) were homogenized with 20% trichloroacetic acid in orthophosphoric acid (2 M) and made up to 50 mL with deionized water. After filtration through Whatman No. 1 paper, 5 mL of filtrate was mixed with 5 mL of 0.005 M 2-thiobarbituric acid (TBA) solution and incubated for 15 h at room temperature in the dark. Absorbance was recorded at 530 nm against a reagent blank (5 mL TBA + 5 mL distilled water), and the results were expressed as TBARS values *i.e*., malonaldehyde (MDA) concentration per kg of the respective samples.^32^

#### 2.13.3. Migration assay

Migration of Ag and Zn from the films into chicken meat was analyzed using inductively coupled plasma mass spectrometry (ICP-MS; Agilent 7850, Agilent Technologies, USA) on days 0, 7, and 14. The meat samples were separated from the films, digested using a microwave digestion system, and diluted appropriately. The mass fraction (*w*) of Ag and Zn in mg kg^−1^ was calculated using the formula:*w* = *a* × *V* × *F m*^−1^where *a* = element concentration in test solution (µg L^−1^), *V* = digestion solution volume (mL), *F* = dilution factor, and *m* = initial sample mass (g).^33^

### 2.14. Statistical analysis

All experiments were performed in triplicate and repeated independently three times. The results are presented as mean ± standard deviation (SD) of three independent experiments. Statistical analyses and data visualization were conducted using GraphPad Prism version 8.2.1 (GraphPad Software Inc., California, USA). A two-way analysis of variance (ANOVA) was applied to evaluate the antioxidant activity of Ag/ZnO–N. For the *in vitro* bacterial killing kinetics and *ex vivo* meat packaging assays, a repeated-measures two-way ANOVA with Geisser–Greenhouse correction was employed to account for unequal variances over time. The safety assay involving commensal gut microflora was analyzed using a one-way ANOVA. Differences were considered statistically significant at *P* ≤ 0.05 and highly significant at *P* ≤ 0.01.

## 3. Results and discussion

### 3.1. Green synthesis of Ag/ZnO NCs

Green synthesis of Ag/ZnO NCs was successfully carried out, using the methanolic extract of *C. longa* as a biogenic reducing and stabilizing agent.^16^ The distinct brown coloration indicated surface plasmon resonance (SPR) typical of metallic NPs, confirming successful synthesis of Ag/ZnO NC.

UV-vis spectrophotometry revealed a broad absorption band at 340–450 nm, corresponding to the SPR peak of Ag within the ZnO matrix. The *C. longa* extract served as a green, non-toxic, and sustainable reductant, with curcuminoids and polyphenols acting as reducing and stabilizing agents. These biomolecules likely adsorbed onto the NC surface, preventing agglomeration, enhancing colloidal stability, and promoting uniform morphology.^16^

### 3.2. Entrapment of Ag/ZnO NCs with cinnamaldehyde

Green-synthesized Ag/ZnO NCs were successfully functionalized by suspending Ag/ZnO NCs in varying concentrations of methanolic cinnamaldehyde.^34^ Phytochemical compounds, such as cinnamaldehyde, are recognized for their potent antibacterial and antioxidant properties and are being explored as eco-friendly food preservatives.^17,35^ However, their industrial application in pure forms in food systems is constrained primarily by volatility, hydrophobicity, and chemical instability, which limit their persistence and uniformity in food matrices. Entrapment within nanostructures enhances solubility, stability, and sustained release, mitigating these limitations and extending antibacterial activity.^12,36^ To identify the optimal formulation for subsequent applications, the Ag/ZnO–N prepared with the lowest cinnamaldehyde concentration exhibiting the highest antibacterial efficacy was selected for further analyses.

### 3.3. *In vitro* antimicrobial activity of Ag/ZnO–N

In this study, irrespective of the tested MDR bacterial strains, the minimum inhibitory concentration (MIC) of the green-synthesized Ag/ZnO NCs was determined to be 31.25 µg mL^−1^, consistent with the earlier findings^16^ (Table 1). Remarkably, following entrapment with cinnamaldehyde, the MIC of Ag/ZnO–N decreased four-fold- from 31.25 to 7.18 µg mL^−1^ (Table 1), indicating a substantial enhancement in antibacterial efficacy.

**Table 1:** MIC values of Ag/ZnO and Ag/ZnO–N against representative strains of MDR bacteria

Concentration of EOs (% [v/v])	MDR bacteria	MIC (µg mL^−1^)	
		Ag/ZnO	Ag/ZnO–N
0.10	*S.* Enteritidis	31.25	7.81
	*S.* Typhimurium	31.25	7.81
	EAEC	31.25	7.81
	MRSA	31.25	7.81
0.25	*S.* Enteritidis	31.25	7.81
	*S.* Typhimurium	31.25	7.81
	EAEC	31.25	7.81
	MRSA	31.25	7.81
0.50	*S.* Enteritidis	31.25	7.81
	*S.* Typhimurium	31.25	7.81
	EAEC	31.25	7.81
	MRSA	31.25	7.81
0.75	*S.* Enteritidis	31.25	7.81
	*S.* Typhimurium	31.25	7.81
	EAEC	31.25	7.81
	MRSA	31.25	7.81
1.00	*S.* Enteritidis	31.25	7.81
	*S.* Typhimurium	31.25	7.81
	EAEC	31.25	7.81
	MRSA	31.25	7.81
2.50	*S.* Enteritidis	31.25	7.81
	*S.* Typhimurium	31.25	7.81
	EAEC	31.25	7.81
	MRSA	31.25	7.81
5.00	*S.* Enteritidis	31.25	7.81
	*S.* Typhimurium	31.25	7.81
	EAEC	31.25	7.81
	MRSA	31.25	7.81
7.50	*S.* Enteritidis	31.25	7.81
	*S.* Typhimurium	31.25	7.81
	EAEC	31.25	7.81
	MRSA	31.25	7.81
10.00	*S.* Enteritidis	31.25	7.81
	*S.* Typhimurium	31.25	7.81
	EAEC	31.25	7.81
	MRSA	31.25	7.81

Among the tested formulations, Ag/ZnO–N prepared using 0.10% methanolic cinnamaldehyde exhibited the highest antimicrobial potential, requiring the least concentration of EO, was therefore selected for subsequent assays. The MIC and MBC values of Ag/ZnO–N were further determined against all MDR test bacteria, wherein the MIC values of Ag/ZnO–N consistently decreased four-fold relative to Ag/ZnO NCs, whereas the MBC values remained unchanged following EO entrapment (Table 2).

**Table 2:** MIC and MBC values of Ag/ZnO–N entrapped with 0.10% of cinnamaldehyde against MDR test bacterial strains

MDR bacteria	Isolate ID	MIC/MBC (µg mL^−1^)	
		Ag/ZnO	Ag/ZnO–N
*S.* Enteritidis	SE1	31.25/62.5	7.81/62.50
	SE2	31.25/62.5	7.81/62.50
	SE3	31.25/62.5	7.81/62.50
*S.* Typhimurium	ST1	31.25/62.5	7.81/62.50
	ST2	31.25/62.5	7.81/62.50
	ST3	31.25/62.5	7.81/62.50
EAEC	E1	31.25/62.5	7.81/62.50
	E2	31.25/62.5	7.81/62.50
	E3	31.25/62.5	7.81/62.50
MRSA	SA1	31.25/62.5	7.81/62.50
	SA2	31.25/62.5	7.81/62.50
	SA3	31.25/62.5	7.81/62.50

The pronounced antibacterial activity of the green-synthesized Ag/ZnO NCs can be attributed primarily to the generation of reactive oxygen species (ROS), which induce oxidative stress and damage cellular components, including lipids, proteins, and nucleic acids. Additionally, the synergistic electrostatic interactions between Ag and ZnO facilitate the rupture of bacterial plasma membranes, compromising cell integrity. Ag^+^ and Zn^2+^ ions further interact with the negatively charged bacterial cell wall and membrane surfaces, disrupting structural stability and metabolic functions.^16^

Cinnamaldehyde, a principal aromatic aldehyde of *Cinnamomum* spp., is widely utilized as a food flavouring and preservative^11^ and exhibits potent antimicrobial activity against *E. coli*,^10,20,37^*S. aureus*^10,37^ and *Salmonella* spp.^20,38^ Although the precise mode of action of EOs remains incompletely understood, their antimicrobial efficacy is generally attributed to multi-target mechanisms rather than a single specific pathway.^17^ The hydrophobic nature of EOs allows their integration into the lipid bilayers of bacterial membranes and mitochondria, increasing membrane permeability and disrupting cell wall structures.^39^ Such interactions cause membrane potential collapse, disruption of proton gradients, leakage of intracellular contents, and coagulation of cytoplasmic material, ultimately leading to cell death. Furthermore, EOs can inactivate essential enzymes required for bacterial growth and survival.^17^

When combined with NPs, the inherent antimicrobial properties of EOs can be significantly enhanced through complementary mechanisms. The EO molecules enhance NP-bacteria interactions, while the NPs facilitate EO delivery across bacterial membranes. This synergistic interaction effectively broadens the antibacterial spectrum and mitigates drug resistance.^7,35,36^ EO-entrapped NCs such as Ag/ZnO–N therefore represent a promising next-generation antimicrobial strategy, wherein enhanced EO diffusion and controlled release kinetics augment bacterial membrane disruption and ROS-mediated killing.^40^

### 3.4. Characterisation of Ag/ZnO–N

**Fig. 2:**
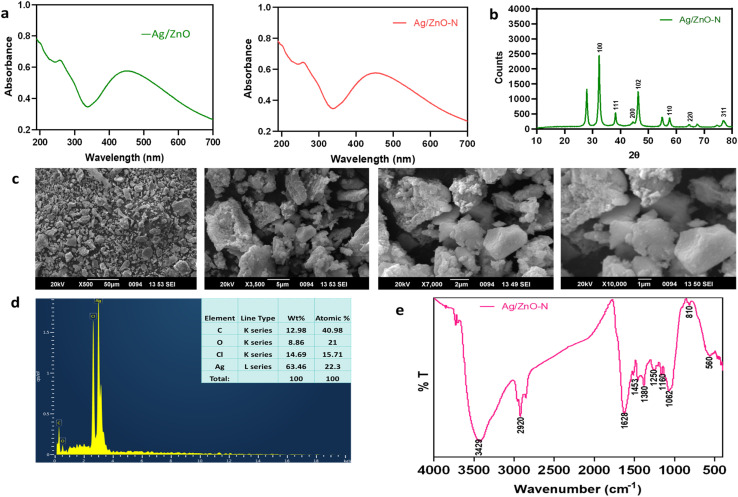
Physicochemical characterization of Ag/ZnO–N. (a) UV-vis spectrum, (b) FTIR spectrum, (c) PXRD pattern, (d) SEM micrograph, and (e) EDAX profile of Ag/ZnO–N.

The UV-vis spectra of Ag/ZnO–N closely resembled those of Ag/ZnO NCs, displaying a broad SPR band between 370 and 475 nm, characteristic of the co-existence of Ag and ZnO and confirming successful nanocomposite formation. An additional absorption region observed between 190 and 300 nm was attributed to phytochemicals originating from the *C. longa* extract used in green synthesis. Since the characteristic absorbance peaks of cinnamaldehyde also occur within this range, its distinct spectral signature was likely masked by the strong absorption of surface-adsorbed polyphenolic compounds on the Ag/ZnO NCs (Fig. 2a).^21^

The FTIR spectral profile of Ag/ZnO–N (Table 3) was analogous to that of Ag/ZnO NCs,^16^ with notable variations corresponding to cinnamaldehyde entrapment. The observed shifts and emergence of additional peaks, particularly in regions associated with –C=O and –C–H stretching vibrations (Table 3), confirm the successful incorporation of cinnamaldehyde onto the surface of Ag/ZnO NCs through physicochemical interactions, indicating effective functionalization (Fig. 2e).

**Table 3:** Functional groups associated with Ag/ZnO–N as per FTIR spectrum

Peaks (cm^−1^)	Functional groups	References
3429	OH	41
2960	CH stretching	42
1628	Aldehyde carbonyl stretching	43
1453	Alcohol OH	44
1380	CH_3_ stretch	34
1250	C–O–C symmetric expansion and phenolic C–OH stretching vibration	45
1160	C=O stretching	46
1062	COOH–Ag interactions	46
810	Aromatic C–H out-of-plane bending	47
560	Metal oxygen bands	16

The PXRD pattern of Ag/ZnO–N closely resembled that of the pristine Ag/ZnO NCs, albeit with discernible variations.^16^ The distinct diffraction peaks at 32.3°, 46.3°, and 57.5° corresponded to the (100), (102), and (110) planes of the hexagonal wurtzite structure of ZnO, while those at 38.2°, 44.3°, 64.6°, and 76.7° were indexed to the (111), (200), (220), and (311) planes of the face-centered cubic structure of metallic Ag, consistent with JCPDS card numbers 89-3722 and 00-036-1451, respectively.^16^ Notably, compared with the Ag/ZnO NCs, the Ag/ZnO–N diffractogram exhibited attenuated peak intensities at 32.3°, 38.2°, 44.3°, 64.6°, and 76.7°, with a pronounced decrease at 38.2° and near disappearance of the 44.3° reflection (Fig. 2b). This attenuation suggests a reduction in crystallinity, possibly due to the surface modification and partial amorphization induced by cinnamaldehyde interaction with the Ag/ZnO lattice.^48^ Such crystallographic alterations further corroborate successful entrapment and surface functionalization of cinnamaldehyde on the matrices of Ag/ZnO NCs.

The surface morphology of Ag/ZnO–N was examined using SEM, which revealed irregularly shaped, polycrystalline structures with evident agglomeration-consistent with the morphology (Fig. 2c) observed for unmodified Ag/ZnO NCs.^16^ The observed agglomeration could be attributed to the high surface energy of NPs and possible cross-linking interactions with cinnamaldehyde during entrapment. Elemental analysis of Ag/ZnO–N using EDAX confirmed the presence of Ag, C, O, and Cl (Fig. 2d). The Ag signal originated from the silver nitrate precursor used in synthesis, while C and O peaks were likely derived from the phytoconstituents of *C. longa* extract and cinnamaldehyde molecules adsorbed on the nanocomposite surface.^49^ The trace detection of Cl could be associated with residual phytochemicals in the plant extract or environmental contamination during sample preparation.^50^ The absence of Zn peaks in the EDAX spectra (Fig. 2d) might either be due to the technique's relatively low elemental detection sensitivity compared with advanced analytical tools such as ICP-MS^51^ or localized absorption of X-ray signals from Zn by the agglomerated material surrounding it.^52^ Overall, SEM-EDAX findings corroborate the morphological and compositional integrity of the Ag/ZnO–N, validating successful synthesis and surface functionalization.

### 3.5. *In vitro* cell viability assay

**Fig. 3:**
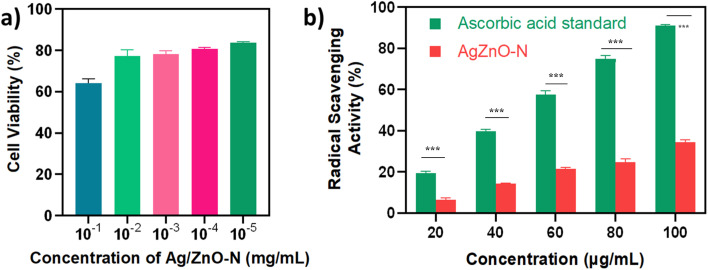
*In vitro* antioxidant and cytotoxicity assays of Ag/ZnO–N. (a) MTT-based cytotoxicity assay and (b) antioxidant activity determined by the ABTS radical scavenging assay.

MTT cytotoxicity assay was conducted to evaluate the *in vitro* safety of Ag/ZnO–N using Vero cell lines. The results indicated a dose-dependent cytotoxic response across the tested concentration range (10^−1^ to 10^−5^ mg mL^−1^). At the highest concentration (10^−1^ mg mL^−1^), Ag/ZnO–N exhibited 64.04 ± 2.07% cell viability (Fig. 3a). In a previous study, Ag/ZnO NCs were observed to be non-haemolytic when tested *in vitro* using chicken RBCs.^49^ The reduced cytotoxicity observed in this study may be attributed to the protective coating of biomolecules associated with Ag/ZnO NCs during green synthesis and subsequent cinnamaldehyde entrapment.^34^ Interestingly, aqueous cinnamon bark extracts exerted cytoprotective effects on cisplatin-treated Vero cells by modulating apoptotic pathways. Similarly, cinnamaldehyde may have mitigated cellular impairments induced by Ag/ZnO NCs, thereby enhancing eukaryotic cell viability. Expanding evaluations across multiple eukaryotic cell lines and *in vivo* systems will further validate the biosafety.

### 3.6. *In vitro* antioxidant activity

Autooxidation is a major cause of food deterioration, leading to nutrient loss, diminished sensory attributes, and reduced consumer acceptance.^17^ Antioxidants-whether synthetic or natural-mitigate oxidative stress by neutralizing free radicals.^53^

In the present study, Ag/ZnO–N exhibited a significant (*P* < 0.001; two-way ANOVA) concentration-dependent free radical scavenging activity (RSA) in the ABTS^˙+^ assay, although its activity was comparatively lower than that of ascorbic acid. At the highest concentration tested (100 µg mL^−1^), Ag/ZnO–N achieved an RSA of 29.39 ± 2.21%, whereas ascorbic acid displayed 91.77 ± 1.18% (Fig. 3b). The antioxidant activity of Ag/ZnO–N is likely attributed to the synergistic effects of green-synthesized Ag/ZnO NCs and entrapped cinnamaldehyde, both of which possess inherent radical scavenging capacity.^15,17^ Moreover, the *C. longa* extract employed for biosynthesis-rich in flavonoids, polyphenols, and ascorbic acid-may further contribute to the overall antioxidant potential of the Ag/ZnO NCs.

The demonstrated biocompatibility and antioxidant potential of Ag/ZnO–N highlight its promise as a safe and functional nanocomposite for active packaging applications.

### 3.7. Preparation of active antimicrobial packaging film

In this study, an alginate polymer matrix crosslinked with calcium chloride and plasticized with glycerol was employed as a vehicle for delivering Ag/ZnO–N into refrigerated chicken meat to enhance preservation. Alginate, a hydrophilic polysaccharide composed of (1,4)-linked β-d-mannuronic and α-l-guluronic acids, is derived from brown algae (*Phaeophyceae*) and widely used as a stabilizing, thickening, and gelling agent in foods. Its non-toxic, biodegradable nature and ability to form transparent, flexible films with good gas-barrier properties make it suitable for packaging applications. However, its limited antimicrobial and light-barrier capacities can be improved by incorporating functional additives such as phytochemicals and NPs.^54^

The physicochemical properties of alginate films depend on the type and concentration of plasticizer and crosslinking agent used.^55^ Crosslinkers modulate the release of active compounds by restricting polymer chain mobility, while also improving mechanical strength, reducing water solubility and swelling, and delaying biodegradation.^56,57^ In this study, food-grade calcium chloride (E509;^58^ was used as the crosslinker, and glycerol served as a non-toxic plasticizer^59^ for film formation.

### 3.8. *In vitro* dose- and time-dependent growth kinetics of MDR-bacterial strains treated with alginate-Ag/ZnO–N films

In this study, MDR bacterial strains co-incubated with alginate film controls and untreated controls displayed a progressive increase in growth over time. In a previous investigation, Ag/ZnO NCs alone exhibited complete inhibition of bacterial growth within 240 minutes at both MIC and MBC.^60^

**Fig. 4:**
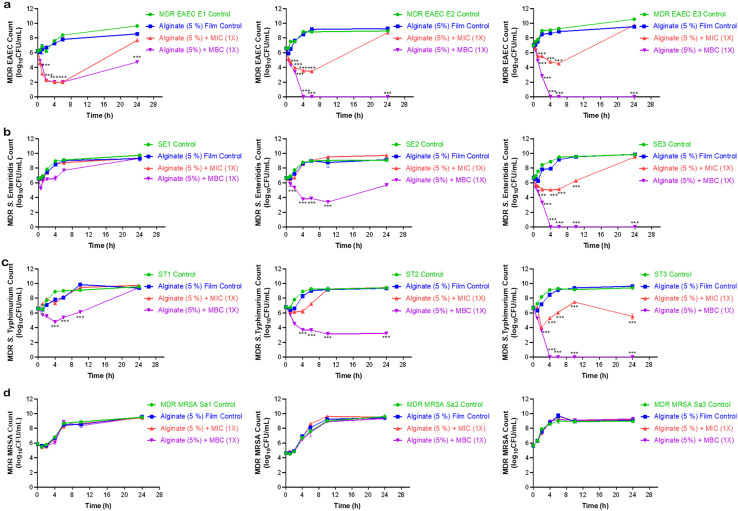
Dose- and time-dependent growth kinetics of MDR bacterial strains treated with alginate-Ag/ZnO–N films. Growth inhibition curves of (a) EAEC, (b) *Salmonella* Enteritidis, (c) *S.* Typhimurium, and (d) MRSA following exposure to alginate-Ag/ZnO–N films.

The present findings demonstrated that alginate-Ag/ZnO–N films exerted *in vitro* dose- and time-dependent antibacterial activity against the tested food-borne pathogens, though the response varied among bacterial strains (Fig. 4a–d). Notably, MRSA isolates demonstrated comparatively lower susceptibility. Films incorporating MBC levels of Ag/ZnO–N exhibited superior antibacterial effects than those loaded at MIC levels. Antimicrobial activity was most pronounced during the initial hours of incubation, with bacterial counts gradually increasing thereafter. Similar transient antibacterial effects were reported earlier with starch-based active packaging films containing nisin, lysozyme, and EDTA, where activity diminished with prolonged exposure. The antimicrobial effects of the tested films against spoilage-causing microorganisms were apparent for short incubation times; however, prolonged co-incubation could not exhibit pronounced antibacterial activity.^61^

### 3.9. Effect of alginate-Ag/ZnO–N film on commensal gut microflora

The gastrointestinal microbiota forms a critical component of the host immune system, providing defense against diverse infections. Given the potential of phytocompounds and NPs in oral delivery systems, it is crucial to assess their impact on gut microbial balance.^34^

**Fig. 5:**
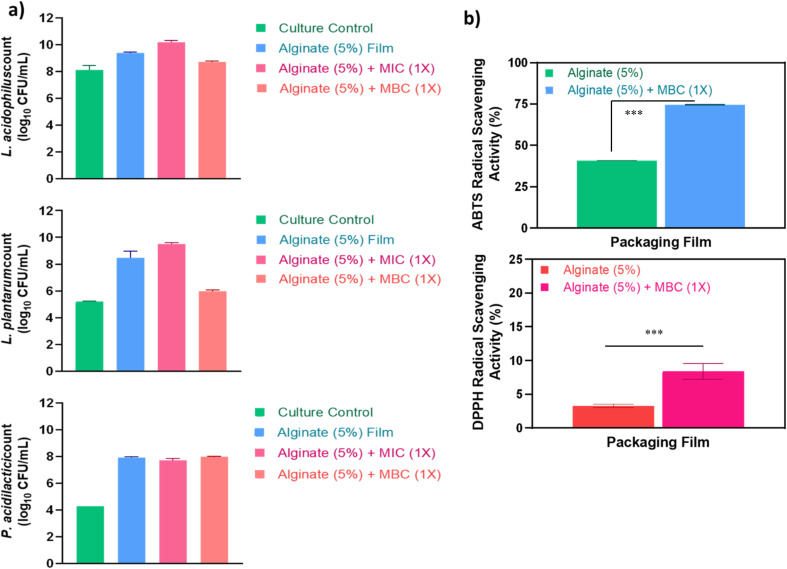
*In vitro* safety and antioxidant potential of alginate-Ag/ZnO–N films. (a) Growth response of commensal gut isolates (*Lactobacillus acidophilus* MTCC 10307, *L. plantarum* MTCC 5690, and *Pediococcus acidilactici* ATCC 8042) upon co-incubation with the films; (b) radical scavenging activity measured by ABTS and DPPH assays.

In the present study, the alginate film, either alone or loaded with MIC and MBC levels of Ag/ZnO–N, exhibited no significant inhibitory effect (*P* > 0.05) on representative commensal gut isolates *L. acidophilus* MTCC 10307, *L. plantarum* MTCC 5690, and *Pediococcus acidilactici* ATCC 8042. Interestingly, an increase in viable counts of these beneficial bacteria was observed upon co-incubation with alginate-based films compared to untreated controls (Fig. 5a). This aligns with previous observations that green-synthesized Ag/ZnO NCs^60^ and Ag–N^21^ exert minimal effects on commensal microbiota.

Moreover, increase in the growth of commensal bacteria may be attributed to the inherent prebiotic potential of alginate, which supports probiotic proliferation.^62^ Edible alginate films have been reported as effective carriers for probiotic bacteria, enhancing their stability and extending the shelf life of foods such as fruits, vegetables, and meat products.^63^ Considering that alginate- Ag/ZnO–N films containing MBC levels of Ag/ZnO–N exhibited superior antibacterial efficacy against pathogens while sparing beneficial gut flora, this formulation was selected for subsequent *ex vivo* analyses, with neat alginate films serving as controls.

### 3.10. Characterisation of alginate-Ag/ZnO–N film

The alginate and alginate-Ag/ZnO–N films (MBC level) were characterized by FTIR, TGA-DTG, and AFM analyses, along with evaluation of OTR and WVTR.

**Fig. 6:**
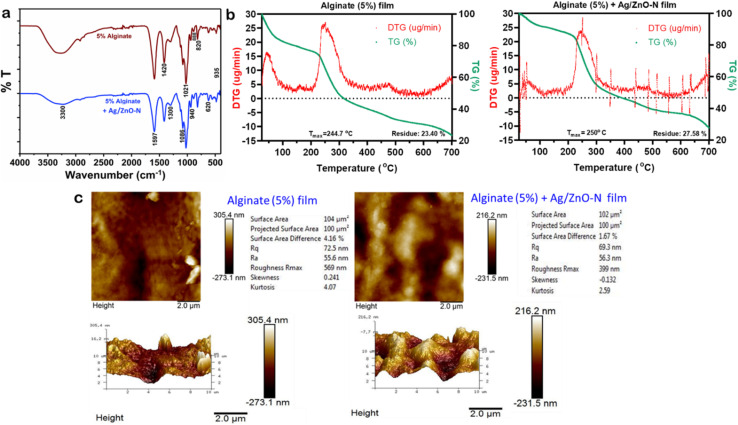
Physicochemical properties of alginate and alginate-Ag/ZnO–N films. (a) FTIR spectra, (b) TGA–DTG thermograms, and (c) AFM images of alginate and alginate-Ag/ZnO–N films.

The FTIR spectra of alginate films displayed characteristic peaks at 1420, 1021, 888, 820, and 935 cm^−1^, whereas the alginate-Ag/ZnO–N films exhibited distinct peaks at 3300, 1597, 1300, 1086, 940, and 620 cm^−1^ (Fig. 6a). The peaks at 888, 820, and 935 cm^−1^ corresponded to vibrations of mannuronic and guluronic acid residues, confirming the structural integrity of the alginate backbone. The absorption at 1086 cm^−1^ was attributed to C–C and C–O stretching, indicative of effective cross-linking within the polymer network. The bands at 1420 and 1597 cm^−1^ were assigned to the symmetric and asymmetric stretching vibrations of carboxylate (COO^−^) groups, suggesting ionic interactions between polymeric carboxyl moieties and divalent calcium ions. The broad band near 3300 cm^−1^ corresponded to O–H stretching vibrations. A notable reduction in the intensity of the 1021 cm^−1^ peak in the alginate-Ag/ZnO–N film, relative to the neat alginate film, suggested weaker interactions of Ca^2+^ from the cross-linking agent with the guluronic acid residues of alginate.^64^ This observation indicates the successful incorporation of nanoadditives within the polymer matrix without inducing major conformational alterations to the calcium alginate structure. Additionally, the enhanced intensities at 3300, 1597, and 1420 cm^−1^ (Fig. 6a) in the nanocomposite film implied the formation of hydrogen bonds between the oxygen moieties of Ag/ZnO–N and the –OH, –COOH, and –CH_3_ groups of alginate,^65^ confirming effective interaction and stabilization of Ag/ZnO–N within the biopolymeric matrix.

The TGA–DTA–DTG thermograms of the neat alginate and alginate-Ag/ZnO–N films revealed broadly similar thermal profiles with discernible differences in stability parameters (Fig. 6b). The degradation of alginate proceeded through a multi-step mechanism: the initial minor weight loss below 100 °C corresponded to the evaporation of physically adsorbed moisture and other volatiles. A pronounced weight loss occurring between 200 and 350 °C was associated with the thermal decomposition of the alginate backbone, specifically the cleavage of glycosidic linkages and volatilization of the glycerol plasticizer. Subsequent weight loss events in the 400–500 °C and 600–700 °C ranges (Fig. 6b) were indicative of the progressive breakdown of carbonaceous intermediates formed during earlier stages of degradation.^66^

The temperature of maximum decomposition (*T*_max_) for the neat alginate film was recorded at 244.7 °C, whereas the alginate-Ag/ZnO–N film exhibited a slightly elevated *T*_max_ of 250 °C. Corresponding maximum mass loss rates were 26.3 µg min^−1^ and 24.14 µg min^−1^, respectively (Fig. 6b), reflecting enhanced thermal stability of the nanocomposite film. This improvement may be attributed to strong interfacial interactions between Ag/ZnO–N and the alginate matrix, which likely restricted polymer chain mobility and delayed thermal decomposition.^67^ Furthermore, the residual char content at 700 °C increased from 23.40% in the neat alginate film to 27.58% in the alginate-Ag/ZnO–N film, representing a 4.18% rise in thermal residue. This higher residue can be ascribed to the presence of inorganic Ag/ZnO, which remained stable after the degradation of the organic matrix.^66^ Sharp fluctuations observed in the DTG trace of the alginate-Ag/ZnO–N film are considered experimental artifacts.^68^

The AFM analysis revealed comparable surface morphologies for alginate and alginate-Ag/ZnO–N films, with subtle variations in topographical parameters. The root mean square roughness (*R*_q_), average roughness (*R*_a_), and maximum roughness depth (*R*_max_) of the alginate film were 72.5, 55.6, and 569 nm, respectively, whereas those of the alginate-Ag/ZnO–N film were 69.3, 56.3, and 399 nm (Fig. 6c). Cross-linking of alginate with Ca^2+^ resulted in pronounced surface irregularities in both film types, as evidenced by AFM images (Fig. 6c). The prominent ‘peaks and valleys’ observed could be attributed to alginate self-aggregation.^64^ However, the incorporation of Ag/ZnO–N did not markedly alter the surface roughness parameters, likely due to favourable interfacial compatibility between the nanofiller and the alginate matrix. This observation contrasts with reports where ZnO NP addition to soybean polysaccharide^69^ or Geranium EO-based polymers^70^ resulted in increased roughness owing to weak additive-polymer interactions.

On assessing the OTR, the film demonstrated very high permeability, with oxygen quickly permeating from the upper to the lower chamber within seconds. Similarly, on assessing the WVTR, the film failed to withstand the testing conditions, indicating high water vapor permeability. Despite their environmental benefits, most biodegradable polymers remain underutilized in barrier packaging due to inadequate thermal and mechanical properties, as well as insufficient oxygen and water vapor barrier capabilities compared to conventional packaging materials.^71^ Further optimization of film barrier properties through appropriate chemical and structural modifications may help overcome these deficiencies. While the developed films demonstrated high oxygen and water vapor permeability, their bifunctional potential, particularly antioxidant capacity, offers a promising avenue for active packaging applications. Hence, the *in vitro* antioxidant activity of the alginate-Ag/ZnO–N film was evaluated to determine its ability to scavenge free radicals and potentially retard oxidative deterioration in foods.

### 3.11. *In vitro* antioxidant activity of alginate-Ag/ZnO–N film

The *in vitro* antioxidant activity of alginate and alginate-Ag/ZnO–N films was evaluated using DPPH and ABTS^˙+^-based free radical scavenging assays. Both films exhibited highly significant (*P* < 0.001) radical scavenging activity; however, the alginate-Ag/ZnO–N film showed superior activity in both assays, with DPPH and ABTS^˙+^ scavenging values of 8.38 ± 1.18% and 74.50 ± 0.14%, respectively, compared to 3.30 ± 0.25% and 40.90 ± 0.11% for the neat alginate film (Fig. 5b).

Ag/ZnO NCs synthesized using *C. longa* extract exhibited dose-dependent antioxidant potential, attributed to bioactive phytochemicals such as flavonoids, tannins, and polyphenols.^15^ These phytoconstituents, along with the entrapped cinnamaldehyde, act as natural antioxidants by donating hydrogen atoms to neutralize free radicals, quenching singlet oxygen species, and chelating pro-oxidant metal ions.^14,39,53^

Interestingly, the alginate matrix itself contributed to the overall antioxidant activity. This is likely due to the chelation of divalent Ca^2+^ within its ‘egg-box-like’ structure, preventing hydroxyl radical generation. Moreover, interactions between alginate and phenolic compounds can enhance metal-reducing capacity, inhibit oxidative enzymes, and improve overall radical-scavenging potential.^72^ The relatively lower DPPH scavenging activity compared to ABTS^˙+^ may be attributed to the hydrophobic methanolic medium of the DPPH assay, which limits the interaction of hydrophilic alginate with active agents.^31,72^

### 3.12. *Ex vivo* meat storage study using alginate-Ag/ZnO–N film

**Fig. 7:**
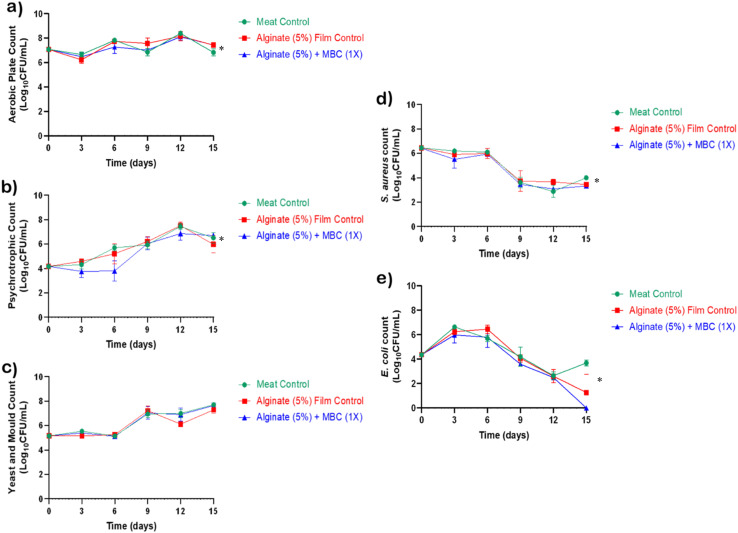
*Ex vivo* meat storage study: microbiological assessment. Changes in (a) aerobic plate count (APC), (b) psychrotrophic bacterial count, (c) yeast and mould count, (d) *S. aureus* count, and (e) *E. coli* count in meat samples during 15 days of chilled storage.

Given the promising antioxidant and antimicrobial potential of the developed alginate-Ag/ZnO–N films, their efficacy was further assessed under *ex vivo* conditions using chicken breast meat stored under chilled vacuum conditions for 15 days. The microbiological parameters assessed at three-day intervals included APC, psychrotrophic bacterial count, yeast and mould count, *Salmonella* spp., *E. coli*, and *S. aureus*. The alginate-Ag/ZnO–N film exhibited a statistically significant (*P* < 0.05) reduction in APC, psychrotrophic bacterial count, *S. aureus*, and *E. coli* compared with the unwrapped control and neat alginate film. None of the tested meat samples demonstrated detectable *Salmonella* spp., while yeast and mould counts did not differ significantly among treatments (Fig. 7a–e).

Microbial spoilage remains the principal cause of post-harvest food wastage despite advances in preservation technologies, including chemical, biological, and non-thermal approaches. Psychrotrophic bacterial species such as *Pseudomonas*, *Enterobacteriaceae*, *Brochothrix*, and *Lactobacillus* spp. are particularly implicated in meat spoilage.^73^ Recent efforts have therefore focused on innovative packaging systems incorporating natural antimicrobials for safer and more sustainable preservation.^74,75^ The present findings confirm that alginate-Ag/ZnO–N films can effectively limit bacterial proliferation in chilled meat systems while maintaining compliance with microbiological standards.

All tested parameters remained within the permissible limits prescribed by the Food Safety and Standards Authority of India^76^ for fresh and chilled meat, as well as the International Commission on Microbiological Specifications for Foods.^77^ Although the initial aerobic bacterial load was relatively high, the film's antimicrobial effect successfully controlled microbial growth throughout the 15 day storage period.

Lipid oxidation, particularly autooxidation of polyunsaturated fatty acids (PUFAs), represents another key factor contributing to meat spoilage. The TBARS assay revealed no detectable levels of MDA in any of the meat samples throughout storage, indicating that oxidative rancidity was effectively prevented. Since MDA formation is a marker of secondary lipid oxidation, and values exceeding 1.50 mg MDA per kg are typically associated with off flavours in chicken meat,^78,79^ the consistently low TBARS values observed here indicate excellent oxidative stability, likely due to the synergistic antioxidant effect of Ag/ZnO–N and the alginate matrix.

Migration analysis using ICP-MS was performed to determine the transfer of Ag^+^ and Zn^2+^ from the packaging material into the meat samples. European Food Safety Authority stipulates a maximum permissible limit of 0.05 mg kg^−1^ of food for Ag^+^ migration. However, Ag^+^ and Zn^2+^ were undetectable in all the meat samples tested, confirming the film's structural stability and biosafety during storage.^80,81^ The absence of detectable migration in this study underscores the safety of the developed packaging material. However, limited Ag^+^ release might also explain the moderate antimicrobial activity observed. Hence, future optimisation of formulation parameters-including Ag/ZnO–N concentration, plasticiser content, and cross-linking conditions-may help achieve an ideal balance between antimicrobial efficacy and metal ion migration.

## 4. Conclusion

This study reports the design of a biodegradable and edible nanostructured biomaterial by integrating green-synthesized Ag/ZnO NCs entrapping cinnamaldehyde into a food-grade alginate matrix composed entirely of U.S. Food and Drug Administration “generally recognized as safe” constituents. The resulting edible biomaterial exhibited strong antibacterial activity against MDR pathogens, demonstrated biocompatibility with eukaryotic cells, and supported beneficial gut microbiota, thereby offering a safe, ingestible, and sustainable food-contact platform. By integrating bio-sourced, renewable constituents with proven antimicrobial functionality, the alginate-Ag/ZnO–N film advances edible packaging strategies aligned with one health principles and circular bioeconomy frameworks. Future investigations focusing on nano-matrix interfacial interactions, controlled release kinetics, barrier mechanics, and degradation pathways will further strengthen the understanding of structure-property-performance relationships and enhance material optimization. In addition, systematic evaluation of long-term exposure effects, food chain interactions, and extended migration behaviour will provide deeper insights into safety and functional longevity. Comprehensive assessment of environmental aspects, including the fate of nanoparticles, their interaction with soil microbiota, and the biodegradability and life-cycle profile of the composite film, will further support its sustainable translation. Collectively, this work contributes to the development of next-generation edible biomaterials that enhance food safety while supporting environmental sustainability and the United Nations SDGs.

## Conflicts of interest

There are no conflicts to declare.

## Data Availability

All the data supporting this article have been included in the main text. The authors can provide data upon request, if any.
